# Feasibility and Acceptability of a Motivational Interviewing–Based Telehealth Intervention for Bacterial Sexually Transmitted Infection Screening: Protocol for a Sequential Explanatory Mixed Methods Study

**DOI:** 10.2196/64433

**Published:** 2024-08-29

**Authors:** Akshay Sharma, Sara Boyd, Erin Elizabeth Bonar

**Affiliations:** 1 Department of Health Behavior and Biological Sciences University of Michigan School of Nursing Ann Arbor, MI United States; 2 Michigan Innovations in Addiction Care through Research and Education Program Department of Psychiatry University of Michigan Ann Arbor, MI United States

**Keywords:** HIV, sexually transmitted infections, gonorrhea, chlamydia, syphilis, telehealth, motivational interviewing, self-testing, sexual health, sexual and gender minorities, mobile phone

## Abstract

**Background:**

Gay, bisexual, and other men who have sex with men living with HIV (GBMSM-LWH) in the United States bear a heavy burden of bacterial sexually transmitted infections (STIs). Timely diagnosis and treatment are key to prevention. Only a few studies have combined home specimen self-collection for bacterial STI screening with live audio and video (AV) conferencing. None have focused on GBMSM-LWH or incorporated motivational interviewing (MI), a client-centered, strengths-based counseling approach that seeks to support individuals to create positive behavioral change.

**Objective:**

Our study seeks to investigate the feasibility and acceptability of an MI-based telehealth intervention that integrates home specimen self-collection from different anatomical sites of possible exposure and MI delivered via live AV conferencing to engage sexually active GBMSM-LWH in bacterial STI screening.

**Methods:**

Participants are being recruited from across the United States via advertising on mobile dating apps and social networking sites and via peer referral. Phase 1 involves piloting the delivery of an innovative telehealth intervention for bacterial STI screening to 75 GBMSM-LWH. Our intervention includes three components: (1) a pretest live AV conferencing session involving an MI-guided discussion to elicit awareness of bacterial STIs; fill any knowledge gaps; bolster the perceived importance of regularly testing for gonorrhea, chlamydia, and syphilis; and build self-efficacy for specimen self-collection; (2) home self-collection and return via mail of a urine sample (for gonorrhea and chlamydia testing), a throat swab (for gonorrhea and chlamydia testing), a rectal swab (for gonorrhea and chlamydia testing), and a finger-stick blood sample (for syphilis testing); and (3) a posttest live AV conferencing session involving an MI-guided discussion to prepare participants for receiving test results and formulate personalized action plans for seeking treatment (if warranted) and repeat testing. Descriptive statistics and progression ratios will be calculated, and potential variations in our intervention’s feasibility and acceptability will be numerically summarized and graphically visualized. Phase 2 involves elucidating attitudes, facilitators, and barriers related to engaging in each intervention component via semistructured in-depth interviews with a purposive subsample of 20 participants who complete progressively smaller subsets of the pretest session, specimen return for bacterial STI testing, and the posttest session. Thematic analysis will be used to identify, analyze, and report patterns in the data. Quantitative and qualitative data will be integrated at the design, methods, interpretation, and reporting levels.

**Results:**

Study procedures were approved by the Institutional Review Board at the University of Michigan in September 2023. Participant recruitment began in April 2024.

**Conclusions:**

Our study will advance multiple goals of the STI National Strategic Plan for the United States for 2021 to 2025, specifically those pertaining to preventing new STIs; accelerating progress in STI research, technology, and innovation; and reducing STI-related health disparities.

**Trial Registration:**

ClinicalTrials.gov NCT06100250; https://www.clinicaltrials.gov/study/NCT06100250

**International Registered Report Identifier (IRRID):**

DERR1-10.2196/64433

## Introduction

### Background

Gay, bisexual, and other men who have sex with men living with HIV (GBMSM-LWH) in the United States bear a heavy burden of bacterial sexually transmitted infections (STIs), such as gonorrhea, chlamydia, and syphilis. National surveillance data from the Centers for Disease Control and Prevention (CDC) indicate that among GBMSM-LWH who presented for HIV medical care in 2022, gonorrhea positivity was greater than and chlamydia positivity was similar to those in GBMSM without HIV or of unknown serostatus [1]. In addition, a higher proportion of GBMSM-LWH were diagnosed with primary or secondary syphilis compared to GBMSM without HIV or of unknown serostatus [1]. Left undiagnosed and untreated, bacterial STIs may lead to serious health complications, such as epididymitis, orchitis, prostatitis, seminal vesiculitis, and proctitis [2-4]. Inflammatory and ulcerative STIs can also facilitate the onward sexual transmission of HIV in the presence of inadequate viral suppression [5-9]. For example, new syphilis infections can increase HIV viral loads and decrease CD4 cell counts, and syphilitic ulcers can aid the passage of HIV [9]. These observations underscore the importance of diagnosing and treating bacterial STIs among GBMSM-LWH in a timely manner.

It is recommended that sexually active GBMSM-LWH be tested for gonorrhea, chlamydia, and syphilis at least annually or more frequently (eg, every 3 to 6 months) if risk behaviors persist or if they or their sex partners have multiple partners [10]. However, bacterial STI screening rates among GBMSM-LWH in the United States are currently suboptimal [11-13]. Gonorrhea and chlamydia infections at pharyngeal and rectal sites are often asymptomatic and are frequently missed [14-16]. Computer-assisted telephone interviews conducted in 2022 with clinics in 6 high-incidence states found that only 57.5% (432/751) offered extraurethral gonorrhea or chlamydia testing, 74.5% (322/432) of which did not offer tests unless their clients requested tests or reported symptoms [17]. Provider-related barriers to offering testing include limited time, the fear of appearing judgmental, discomfort around discussing sexual risk behaviors, and uncertainty about current screening guidelines [18,19]. Patient-related barriers include low risk perceptions of acquiring bacterial STIs, being unaware of the importance of triple-site gonorrhea and chlamydia testing, the fear of stigmatization by providers, and concerns about privacy and confidentiality [20-22]. Therefore, novel approaches are needed to reduce the impediments to bacterial STI screening experienced by GBMSM-LWH.

Home specimen self-collection has increasingly been used to test for bacterial STIs in studies conducted with diverse populations [23-30]. Self-collected specimens for bacterial STI screening have been shown to be as valid and reliable as those collected by a clinician [31-35]. Telehealth has also demonstrated promise in managing mental health [36-38] and increasing antiretroviral therapy (ART) adherence in people living with HIV [39-43]. Only a few studies have combined home specimen self-collection with live audio and video (AV) conferencing, all of which excluded people living with HIV [28-30]. None have focused on GBMSM-LWH or incorporated motivational interviewing (MI), a client-centered, strengths-based counseling approach that seeks to support individuals to create positive behavioral change [44]. Brief (eg, 30-45 minutes) in-person MI sessions have proven effective in reducing sexual risk behaviors, substance use, and ART nonadherence in people living with HIV [45-47]. Integrating home specimen self-collection from different anatomical sites of possible exposure with MI delivered via live AV conferencing might offer a unique solution to engage GBMSM-LWH in bacterial STI screening. MI-guided discussions have the potential to increase a participant’s knowledge of bacterial STIs when key knowledge gaps exist, enhance their intrinsic motivation to protect themselves and their sex partners, build their self-efficacy for specimen self-collection, and mitigate their barriers to seeking treatment (if warranted) and repeat testing.

### Objectives

To investigate the feasibility and acceptability of an MI-based telehealth intervention to engage GBMSM-LWH in bacterial STI screening, we are conducting a sequential explanatory mixed methods study titled Zenyth. Our intervention includes three components: (1) a pretest live AV conferencing session involving an MI-guided discussion to elicit awareness of bacterial STIs; fill any knowledge gaps; bolster the perceived importance of regularly testing for gonorrhea, chlamydia, and syphilis; and build self-efficacy for specimen self-collection; (2) home self-collection and return via mail of a urine sample (for gonorrhea and chlamydia testing), a throat swab (for gonorrhea and chlamydia testing), a rectal swab (for gonorrhea and chlamydia testing), and a finger-stick blood sample (for syphilis testing); and (3) a posttest live AV conferencing session involving an MI-guided discussion to prepare participants for receiving test results and formulate personalized action plans for seeking treatment (if warranted) and repeat testing. The purpose of this manuscript is to describe the protocol for our study. Our study meets the definition of a National Institutes of Health clinical trial and has been registered in ClinicalTrials.gov (NCT06100250).

## Methods

### Study Overview

Our sequential explanatory mixed methods study includes a quantitative strand (phase 1) followed by a qualitative strand (phase 2). Phase 1 involves piloting the delivery of an innovative telehealth intervention for bacterial STI screening to 75 GBMSM-LWH in the United States, including a pretest MI-guided live AV conferencing session; home self-collection and return of specimens for gonorrhea, chlamydia, and syphilis testing; and a posttest MI-guided live AV conferencing session. For pilot studies, sample sizes between 25 and 150 have been recommended to examine the practicality of the methods to be used in a subsequent larger study [48,49]. Phase 2 involves elucidating attitudes, facilitators, and barriers related to engaging in each intervention component via semistructured in-depth interviews with a purposive subsample of 20 participants who complete progressively smaller subsets of the pretest session, specimen return for bacterial STI testing, and the posttest session. For in-depth interviews, sample sizes between 10 and 30 have been recommended to reach information power and thematic saturation [50,51].

### Theoretical Foundation

Our intervention is guided by the constructs of the information-motivation-behavioral (IMB) skills model of Fisher et al [52]. The model theorizes that information about a behavior (ie, knowledge), motivation to perform the behavior (based on attitudes and social norms), and behavioral skills (ie, self-efficacy and action planning) act collectively to influence the behavior. The extent to which GBMSM-LWH are informed of the importance of regularly screening for gonorrhea, chlamydia, and syphilis; are motivated to act on their knowledge; and have the behavioral skills to self-collect specimens or seek testing could influence their engagement in bacterial STI screening. MI is consistent with the IMB skills model, and MI-guided discussions have the potential to increase a participant’s knowledge of bacterial STIs when key knowledge gaps exist, enhance their intrinsic motivation to protect themselves and their sex partners, build their self-efficacy for specimen self-collection, and mitigate their barriers to seeking treatment (if warranted) and repeat testing. Our choice of measures in the baseline and satisfaction surveys for phase 1 has also been guided by the IMB model. In addition, the theoretical framework of acceptability of health interventions proposed by Sekhon et al [53] has been used to develop the semistructured in-depth interview guide for phase 2.

### Ethical Considerations

Study procedures outlined in this manuscript have been reviewed and approved by the Institutional Review Board at the University of Michigan (HUM00240181) and have been deemed to pose no more than minimal risk to participants.

### Participant Recruitment

Given the common use of social media by GBMSM to find sex partners [54-56], our primary recruitment strategy involves advertising on mobile dating apps (eg, Grindr [Grindr Inc] and Adam4Adam [A4A Network Inc]) and social networking sites (eg, Facebook [Meta Platforms, Inc] and Instagram [Meta Platforms, Inc]). Our goal is to enroll 75 GBMSM-LWH from across the United States to receive the intervention, with approximately 20 (27%) Hispanic participants, 25 (33%) non-Hispanic Black participants, 25 (33%) non-Hispanic White participants, and 5 (7%) participants of other races or ethnicities, mirroring the current racial and ethnic distribution of GBMSM-LWH in the United States [57]. Our advertisements include the study’s logo; the University of Michigan’s logo; images of racially and ethnically diverse men holding hands, cuddling, or kissing; and call-to-action text.

Individuals who click on the advertisements are directed to our study’s landing page programmed in Qualtrics (Qualtrics International, Inc), which provides a brief overview of the intervention. Those who are not interested can exit by closing their browser. Those who are interested can click on a link to the informed consent form that includes a detailed description of all study activities, including completing an eligibility screener; providing contact information to receive study communications and a specimen self-collection box if eligible; completing a baseline survey, each intervention component, and a satisfaction survey in phase 1; and participating in an in-depth interview in phase 2 if selected. Individuals have the option to download a copy of the informed consent form for their records. As they are using their own computers, tablets, or smartphones, informed consent is obtained by them clicking on “I understand what this research study is about, and my questions so far have been answered. I agree to participate in this study” or “I do not wish to participate in this research study.” The consent response, date, and IP address are recorded in Qualtrics. Those who do not consent are directed to a terminal page with a link to the CDC’s website with information and resources on STIs.

Individuals who consent proceed to an eligibility screener that assesses whether they (1) identify as a man (regardless of sex assigned at birth); (2) reside in a US state or territory; (3) will be physically located in a US state or territory when completing study activities; (4) are aged ≥18 years; (5) are of legal age to provide consent for research participation in their US state or territory of residence; (6) have been diagnosed with HIV; (7) have had any kind of condomless sex with ≥2 men in the past year; (8) are willing to provide their contact information (full name, email address, mobile phone number, and mailing address); (9) are able to participate in live AV conferencing sessions using an internet-connected device; and (10) are willing to receive a box that contains kits to self-collect a urine sample, a throat swab, a rectal swab, and a blood sample for bacterial STI testing. Those who do not meet the criteria are directed to a terminal page with a link to the CDC’s website with information and resources on STIs.

Individuals who meet the criteria proceed to a contact information form that asks them to provide their full name, email address, and mobile phone number to receive study communications and their mailing address to receive a specimen self-collection box. Those who do not provide their contact information are directed to a terminal page with a link to the CDC’s website with information and resources on STIs. Those who provide their contact information are informed that a study team member will reach out to verify their full name, email address, mobile phone number, and mailing address. Those who do not provide valid contact information are excluded from further consideration.

Given the reliance on social networks as sources of support by GBMSM, particularly those living with HIV [58-60], our secondary recruitment strategy involves peer referral. Individuals are requested to share the link to our study’s landing page with anyone in their social network who they believe might be interested in participating. The link is provided at the end of the contact information form and on the terminal page. To minimize the potential for fraudulent activity, we use a Completely Automated Public Turing test to tell Computers and Humans Apart (CAPTCHA) challenge, exclude multiple submissions from the same IP address, and review IP addresses to ensure that they are located within the United States.

### Phase 1 Procedures

#### Baseline Survey

Individuals whose contact information has been verified are assigned a randomly generated participant identification number and sent a link to the baseline survey programmed in Qualtrics that includes questions on the following: (1) sociodemographic characteristics (eg, age, race, ethnicity, educational level, employment status, income level, and relationship status); (2) bacterial STI–related knowledge (using a 22-item scale to assess knowledge of gonorrhea, chlamydia, and syphilis [61]); (3) awareness and use of home STI tests and commercial telehealth services (using binary measures of whether one has heard of and used home STI tests sold on the web by companies and commercial telehealth services that offer STI treatment); (4) attitudes around safer sex (using a 13-item subscale from the Sexual Risks Scale [62]); (5) perceived risk of bacterial STIs (using a risk perception ruler ranging from 1 [extremely unlikely] to 10 [extremely likely] to assess the perceived likelihood of contracting a bacterial STI in the next 12 months [61]); (6) safer sex self-efficacy (using a 7-item scale to assess confidence in practicing safer sex [63]); (7) specimen self-collection self-efficacy (using 5-point Likert items to assess the perceived ease of self-collecting each type of specimen); (8) history of HIV testing and management, including the timing of the first positive HIV test, the location of the test, the receipt of HIV medical care, ART use, HIV viral load, and CD4 count [64-66]; (9) history of bacterial STI testing and management, including the frequency of testing for gonorrhea, chlamydia, and syphilis; the timing of the latest tests; the location of the latest tests; the types of specimens provided; test results; and the receipt of treatment; (10) relationship and partner characteristics, including relationship duration and type; partner’s HIV status and use of ART or pre-exposure prophylaxis; and partner’s history of testing for gonorrhea, chlamydia, and syphilis; (11) sexual behaviors, including engagement in oral, anal, and vaginal or frontal sex in the past 12 months and condom use; and (12) history of tobacco use, alcohol use, prescription medication misuse, and other substance use [67].

The baseline survey takes approximately 30 minutes to complete, and it allows participants to skip questions or indicate that they prefer not to answer a question. If participants do not complete the baseline survey within 1 week, reminders are sent at regular intervals over the next 7 weeks. Those who do not complete the baseline survey by the end of 8 weeks are not contacted any further (unless they request an extension). Those who complete the baseline survey receive an Amazon e-gift card (Amazon.com, Inc) worth US $40 and are considered “fully enrolled” participants (ie, participants who proceed to receive the intervention).

#### Intervention Component 1: Pretest MI-Guided Live AV Conferencing Session

Once participants complete the baseline survey, they are contacted to schedule a 30-minute pretest session to be conducted over Zoom (Zoom Video Communications, Inc) with an interventionist trained in MI and on the transmission, prevention, and treatment of bacterial STIs. Time slots are offered in all US time zones and are flexible to meet their individual circumstances. If participants do not schedule a session within 1 week, reminders are sent at regular intervals over the next 7 weeks. Once a time is confirmed, participants are sent a password-protected Zoom link. They are advised to join the session from a private location and are offered the option to reschedule if the original time no longer works or if they anticipate being in a situation where their privacy may be compromised.

Pretest sessions are conducted from a private location, and participants have the option to switch off their own video (ie, use only audio from their end) if they prefer. Sessions are audio recorded using a digital voice recorder to allow for the continuous monitoring of intervention fidelity. No audio or video is recorded in Zoom. The session begins with a conversation to initiate a collaborative partnership and build rapport (MI process: engaging). Next, the interventionist elicits participants’ awareness of the epidemiology and transmission of bacterial STIs among GBMSM-LWH, provides new information to fill any knowledge gaps or address misconceptions around triple-site gonorrhea and chlamydia or syphilis testing, and elicits reactions to this new information. Open-ended questioning and reflective listening are used to gain a better understanding of participants’ perspectives on their own sexual risk and protective behaviors (MI process: focusing). Subsequently, the interventionist explores the participant’s history of bacterial STI testing, their motivations for testing or reasons for not testing, and their perceived benefits and challenges of regular testing (MI process: evoking). To build self-efficacy for specimen self-collection, the interventionist displays the contents of the specimen self-collection box, reviews step-by-step instructions for self-collecting each type of specimen, and addresses any questions or concerns. Participants who express an intent to test with their own provider or at a clinic are encouraged to pursue that route. The session ends by summarizing and affirming the participants’ strengths and goals with respect to protecting their own and their partners’ sexual health and discussing their plans to test for gonorrhea, chlamydia, and syphilis over the next month (MI process: planning).

Participants who do not schedule or attend the pretest session by the end of 8 weeks are deemed not interested in this intervention component (unless they reschedule). Those who complete the session proceed to the next intervention component. Although participants who express an intent to test with their own provider or at a clinic are encouraged to pursue that route, they still proceed to the next intervention component (ie, they are shipped a specimen self-collection box) upon completing the pretest session unless they choose to withdraw from the study.

#### Intervention Component 2: Home Self-Collection and Return of Specimens

Once participants complete the pretest session, they are shipped a box in plain, unmarked packaging that contains kits to self-collect a urine sample, a throat swab, a rectal swab, and a finger-stick blood sample. Boxes are assembled using specimen self-collection kits supplied by the Clinical Virology Research Laboratory at Emory University and shipped using United Parcel Service. The shipment and return statuses of boxes are tracked by a study team member on a regular basis. Each component of the box is described in Textbox 1.

**Description of the box components.**

**Components and description**

1. General information sheet: this sheet provides participants with a brief description of the box contents and information on how to package and deliver their self-collected specimens to the Clinical Virology Research Laboratory (CVRL) at Emory University. It also instructs participants not to include any sort of personally identifiable information in the laboratory requisition form.
2. Urine sample self-collection kit: this kit includes materials for participants to self-collect a urine sample of 3 milliliters (which is <1 teaspoon of urine). These materials include a collection cup, a transfer pipette, and a transport tube containing a buffer solution. The transport tube is affixed with a unique specimen identification number to document gonorrhea and chlamydia test results. The kit also includes written instructions with color images and a QR code that leads to video instructions.
3. Throat swab self-collection kit: this kit includes materials for participants to self-collect a throat swab. These materials include a sterile specimen collection swab and a transport tube containing a buffer solution. The transport tube is affixed with a unique specimen identification number to document gonorrhea and chlamydia test results. The kit also includes written instructions with color images and a QR code that leads to video instructions.
4. Rectal swab self-collection kit: this kit includes materials for participants to self-collect a rectal swab. These materials include a sterile specimen collection swab, 2 lubricant packets, and a transport tube containing a buffer solution. The transport tube is affixed with a unique specimen identification number to document gonorrhea and chlamydia test results. The kit also includes written instructions with color images and a QR code that leads to video instructions.
5. Finger-stick blood sample self-collection kit: this kit includes materials for participants to self-collect a finger-stick blood sample of 500 microliters (which is equivalent to 10 drops of blood). These materials include an alcohol wipe, 2 safety lancets, 2 gauze pads, 2 bandages, and a transport tube containing a buffer solution. The transport tube is affixed with a unique specimen identification number to document syphilis test results. The kit also includes written instructions with color images and a QR code that leads to video instructions.
6. Plastic biohazard bag: this bag is used by participants to enclose the transport tubes containing their self-collected specimens.
7. Laboratory requisition form: this form is used by participants to write down the date on which they self-collected their specimens and the unique specimen identification numbers affixed to the transport tubes.
8. Prepaid shipping box with a category B sticker: this cardboard box affixed with a prepaid United Parcel Service shipping label is used by participants to deliver their self-collected specimens and the completed laboratory requisition form to the CVRL at Emory University.

Participants are requested to self-collect their specimens, enclose the transport tubes in the plastic biohazard bag, complete the laboratory requisition form, place the bag and the form in the prepaid shipping box, and return the box to the Clinical Virology Research Laboratory at Emory University. Specimen return is voluntary, and participants can choose to return all, some, or none of the 4 specimens. However, if no specimens have been received within 3 weeks of box delivery, reminders are sent at regular intervals over the next 5 weeks. Urine samples, throat swabs, and rectal swabs are screened for gonorrhea and chlamydia, and finger-stick blood samples are screened for syphilis. Laboratory personnel can connect the participants’ test results to only the unique specimen identification numbers affixed to the different transport tubes. They do not have access to either the participants’ identification numbers or the participants’ contact information (full name, email address, mobile phone number, and mailing address). Gonorrhea, chlamydia, and syphilis test results are shared with us via a secure Dropbox folder.

Participants who do not deliver any specimens by the end of 8 weeks are deemed not interested in this intervention component (unless they request an extension). Those who deliver some or all specimens proceed to the next intervention component.

#### Intervention Component 3: Posttest MI-Guided Live AV Conferencing Session

Once we receive participants’ test results, they are contacted to schedule a 30-minute posttest session to be conducted over Zoom with the same interventionist who conducted the pretest session. Time slots are offered in all US time zones and are flexible to meet their individual circumstances. If participants do not schedule a session within 1 week, reminders are sent at regular intervals over the next 7 weeks. Once a time is confirmed, participants will be sent a password-protected Zoom link. They are advised to join the session from a private location and are offered the option to reschedule if the original time no longer works or if they anticipate being in a situation where their privacy may be compromised.

Posttest sessions are conducted from a private location, and participants have the option to switch off their own video (ie, use only audio from their end) if they prefer. Sessions are audio recorded using a digital voice recorder to allow for the continuous monitoring of intervention fidelity. No audio or video is recorded in Zoom. The session incorporates the 4 MI processes of engaging, focusing, evoking, and planning. It begins with a conversation to initiate a collaborative partnership and build rapport. The interventionist discusses the meaning of negative and positive bacterial STI test results and assesses participants’ emotional responses to each possible outcome using open-ended questioning and reflective listening. Test results are delivered by screen sharing the laboratory test result form. For participants receiving negative gonorrhea, chlamydia, and syphilis test results, the interventionist uses affirmations to acknowledge their engagement in protective behaviors and jointly formulates a plan to prevent the acquisition of bacterial STIs. For participants receiving a positive gonorrhea, chlamydia, or syphilis test result, the interventionist offers emotional support, discusses the benefits of timely antibiotic treatment, and jointly formulates a linkage to a care plan. Barriers to accessing treatment (eg, the lack of insurance, limited personal transportation, and reluctance to visit one’s own provider) are elicited, and the interventionist works with participants to find practical solutions. For example, if someone lacks insurance, screen sharing is used to give information on local STI clinics that provide free or low-cost services identified using site locators on the CDC and Planned Parenthood websites. If someone has limited personal transportation, the interventionist assists in creating a commute plan that uses public transportation options. If someone is reluctant to visit their own provider, the interventionist assists in finding other STI clinics or identifying commercial telehealth services that offer STI treatment (eg, GoodMDs, PlushHealth, and CallonDoc). Participants are encouraged to notify their sex partners of their positive test results so that those individuals can seek testing. Open-ended questioning and reflective listening are used to engage them in a discussion on sexual risk reduction. Next, the elicit-provide-elicit approach is used to find out what participants already know about the current national recommendations for bacterial STI testing among GBMSM-LWH and to fill any knowledge gaps or address misconceptions around the recommended frequency of testing. The interventionist also explores whether and how home STI tests sold on the web by companies (eg, NURX, myLAB Box, and LetsGetChecked) might fit into the participants’ testing routines. The session ends by reviewing the participants’ personalized action plans formulated based on their test results and summarizing and affirming their strengths and goals with respect to reducing sexual risk behaviors and regularly testing for bacterial STIs. After the session, a copy of the laboratory test result form is shared with participants via a secure Dropbox folder that is accessible only to them and the study team members. The folder also includes a supplemental information sheet on using site locators on the CDC and Planned Parenthood websites to identify local STI clinics to seek treatment (if warranted) and repeat testing. The link to the folder expires after 4 weeks, following which the folder is deleted.

For participants receiving a positive gonorrhea, chlamydia, or syphilis test result, a study team member reviews their state and local statutory reporting requirements and procedures and makes up to 3 attempts to notify the relevant public health authorities. This notification may result in participants being contacted for follow-up and possible contact tracing. A study team member also follows up via phone at 2 weeks and 4 weeks to attempt to determine participants’ treatment statuses. During each of these interactions, the study team member completes a case report form programmed in Qualtrics, documenting whether the participant has initiated treatment, completed treatment, has not yet initiated treatment but has made an appointment with a provider, or has not yet made an appointment with a provider. Those who have not yet made an appointment with a provider are encouraged to do so as soon as possible and offered additional assistance.

Participants who do not schedule or attend the posttest session by the end of 8 weeks are deemed not interested in this intervention component (unless they reschedule). However, a copy of the laboratory test result form and the supplemental information sheet on using site locators to identify local STI clinics is shared with these participants via a secure Dropbox folder that is accessible only to them and the study team members. The link to the folder expires after 4 weeks, following which the folder is deleted.

#### Satisfaction Survey

Upon the completion of intervention delivery, each fully enrolled participant (regardless of their level of engagement in the pretest session, specimen return for bacterial STI testing, and the posttest session) is sent a link to the satisfaction survey programmed in Qualtrics that includes questions on the following: (1) pretest live AV conferencing session experience (using a 4-item subscale from the Telehealth Usability Questionnaire on the quality of interaction with the interventionist [68]; the 12-item Counselor Rating Form short version to assess perceptions of the interventionist [69]; and 5-point Likert items to assess the satisfaction with, willingness to repeat, and likelihood of recommending the session); (2) urine sample self-collection experience (using 5-point Likert items to assess the experience with, willingness to repeat, and likelihood of recommending self-collection [70]); (3) throat swab self-collection experience (using 5-point Likert items to assess the experience with, willingness to repeat, and likelihood of recommending self-collection [70]); (4) rectal swab self-collection experience (using 5-point Likert items to assess the experience with, willingness to repeat, and likelihood of recommending self-collection [70]); (5) finger-stick blood sample self-collection experience (using 5-point Likert items to assess the experience with, willingness to repeat, and likelihood of recommending self-collection [70]); (6) specimen packaging and return experience (using 5-point Likert items to assess the experience with packaging and delivering specimens); (7) posttest live AV conferencing session experience (using a 4-item subscale from the Telehealth Usability Questionnaire on the quality of interaction with the interventionist [68]; the 12-item Counselor Rating Form short version to assess perceptions of the interventionist [69]; and 5-point Likert items to assess the satisfaction with, willingness to repeat, and likelihood of recommending the session); (8) bacterial STI–related knowledge (using a 22-item scale to assess knowledge of gonorrhea, chlamydia, and syphilis [61] for comparison with the baseline survey responses); and (9) the likelihood of testing for bacterial STIs in the future (using 5-point Likert items to assess the likelihood of testing for bacterial STIs at least annually and likelihood of using home STI tests sold on the web by companies).

The satisfaction survey takes approximately 15 minutes to complete, and it allows participants to skip questions or indicate that they prefer not to answer a question. If participants do not complete the satisfaction survey within 1 week, reminders are sent at regular intervals over the next 7 weeks. Those who do not complete the satisfaction survey by the end of 8 weeks are not contacted any further (unless they request an extension). Those who complete the satisfaction survey receive an Amazon e-gift card worth US $20.

### Phase 2 Procedures

To gain a deeper understanding of attitudes, facilitators, and barriers related to engaging in each component of the intervention, we will conduct in-depth interviews with a subsample of 20 participants. Purposive sampling will be used to select a mix of participants who complete progressively smaller subsets of the pretest session, specimen return for bacterial STI testing, and the posttest session [71].

Participants will be contacted to schedule a 1-hour interview with a study team member to be conducted over Zoom or phone (depending on their preference). Time slots will be offered in all US time zones and will be flexible to meet their individual circumstances. If participants do not schedule an interview within 1 week, reminders will be sent at regular intervals over the next 7 weeks. Participants who prefer being interviewed over Zoom will be sent a password-protected Zoom link. They will be advised to join the interview from a private location and be offered the option to reschedule if the original time no longer works or if they anticipate being in a situation where their privacy may be compromised.

Interviews will be conducted from a private location, and participants on Zoom will have the option to switch off their own video (ie, use only audio from their end) if they prefer. Interviews will be audio recorded using a digital voice recorder to allow for verbatim transcription for future analyses. No audio or video will be recorded in Zoom. During the interview, the study team member will ask participants open-ended questions using a semistructured in-depth interview guide. Informed by the theoretical framework of acceptability of health interventions [53], the interviews will focus on a range of participants’ experiences, including factors influencing their attendance (eg, discussing specimen self-collection procedures and receiving bacterial STI test results) or nonattendance (eg, concerns about session length and scheduling conflicts) of the pretest or posttest session and reasons for delivering specimens (eg, confirm bacterial STI status and limited access to testing) or choosing not to deliver specimens (eg, lack of confidence and preference for clinic-based testing).

Participants who do not schedule or attend the interview by the end of 8 weeks will be deemed not interested (unless they reschedule). Those who attend the interview will receive an Amazon e-gift card worth US $40.

### Study Outcomes

#### Primary Outcomes

The feasibility of our intervention will be assessed by documenting the following: (1) the number of participants who schedule a pretest session, (2) the number of participants who join the pretest session within 30 minutes of the start time, (3) the number of participants who deliver each type of specimen within 6 weeks of box delivery, (4) the number of participants who provide specimens of adequate quality for laboratory testing, (5) the number of participants who schedule a posttest session, and (6) the number of participants who join the posttest session within 30 minutes of the start time. Data on these outcomes will be extracted from the participants’ study records.

The acceptability of our intervention will be assessed by documenting the following: (1) overall intervention satisfaction, (2) interventionist perceptions, (3) usability of the pretest and posttest sessions, (4) willingness to repeat the intervention, and (5) likelihood of recommending the intervention to friends or sex partners. Data on these outcomes will be summarized using participants’ responses on the satisfaction survey. Further details are presented in Textbox 2.

**Description of the primary outcomes to assess the acceptability of the intervention.**

**Overall intervention satisfaction**

- Participants’ satisfaction with the pretest session, urine sample self-collection, throat swab self-collection, rectal swab self-collection, finger-stick blood sample self-collection, and the posttest session will be assessed using six 5-point Likert items included in the satisfaction survey [70]. Response values will be summed to obtain a total score ranging from 6 to 30, with higher scores indicating greater overall intervention satisfaction.

**Interventionist perceptions**

- Participants’ perceptions of the interventionist conducting the pretest and posttest sessions will be assessed using two 12-item Counselor Rating Form short versions included in the satisfaction survey [69]. Response values will be summed to obtain a total score ranging from 24 to 168, with higher scores indicating more positive interventionist perceptions.

**Usability of the pretest and posttest sessions**

- Participants’ usability of the pretest and posttest sessions will be assessed using two 4-item subscales from the Telehealth Usability Questionnaire on the quality of interactions with the interventionist during each session included in the satisfaction survey [68]. Response values will be summed to obtain a total score ranging from 8 to 56, with higher scores indicating greater usability of the pretest and posttest sessions.

**Willingness to repeat the intervention**

- Participants’ willingness to repeat the pretest session, urine sample self-collection, throat swab self-collection, rectal swab self-collection, finger-stick blood sample self-collection, and the posttest session will be assessed using six 5-point Likert items included in the satisfaction survey [70]. Response values will be summed to obtain a total score ranging from 6 to 30, with higher scores indicating greater willingness to repeat the intervention.

**Likelihood of recommending the intervention to friends or sex partners**

- Participants’ likelihood of recommending the pretest session, urine sample self-collection, throat swab self-collection, rectal swab self-collection, finger-stick blood sample self-collection, and the posttest session to friends or sex partners will be assessed using six 5-point Likert items included in the satisfaction survey [70]. Response values will be summed to obtain a total score ranging from 6 to 30, with higher scores indicating a greater likelihood of recommending the intervention to friends or sex partners.

#### Secondary Outcomes

The potential impact of our intervention on IMB skills model constructs will be assessed by documenting the following: (1) improvement in bacterial STI–related knowledge, (2) likelihood of testing for bacterial STIs at least annually, and (3) improvement in self-efficacy for specimen self-collection. Data on these outcomes will be summarized using participants’ responses on the baseline and satisfaction surveys. Further details are presented in Textbox 3.

**Description of the secondary outcomes to assess the potential impact of the intervention on information-motivation-behavioral skills model constructs.**

**Improvement in bacterial sexually transmitted infection (STI)–related knowledge**

- Potential increases in participants’ knowledge of gonorrhea, chlamydia, and syphilis will be assessed by comparing responses to the same set of 22 items included in the baseline and satisfaction surveys [61]. Response values will be summed to obtain separate total scores ranging from 0 to 22, with higher scores indicating greater STI-related knowledge.

**Likelihood of testing for bacterial STIs at least annually**

- Participants’ likelihood of testing for bacterial STIs at least annually will be assessed using a single 5-point Likert item included in the satisfaction survey. Response values will range from 1 to 5, with higher values indicating a greater likelihood of testing for bacterial STIs at least annually.

**Improvement in self-efficacy for specimen self-collection**

- Potential increases in participants’ self-efficacy for urine sample self-collection, throat swab self-collection, rectal swab self-collection, and finger-stick blood sample self-collection will be assessed by comparing responses to similar sets of four 5-point Likert items included in the baseline and satisfaction surveys. Response values will be summed to obtain separate total scores ranging from 4 to 20, with higher scores indicating greater self-efficacy for specimen self-collection.

The prevalence of bacterial STIs and receipt of treatment will be assessed by documenting the following: (1) the number of participants who test negative or positive for gonorrhea, chlamydia, and syphilis in our test using our specimen self-collection kits, in a test with their own provider, or in a test at a clinic and (2) the number of participants who initiate treatment with their own provider, at a clinic, or via commercial telehealth services within 1 week of receiving a positive test result. Data on these outcomes will be extracted from the participants’ study records.

### Analytic Plan

Descriptive statistics (eg, means, medians, and ranges for continuous variables and counts and proportions for categorical variables) and progression ratios (ie, proportions of participants who sequentially progress through different intervention components) will be calculated using software for quantitative data analysis (eg, SAS [IBM Corp]). As our study is inherently exploratory, we do not plan on using probability-based statistical inference techniques in line with current best practices [72,73]. Instead, potential variations in our intervention’s feasibility and acceptability across selected participant characteristics and their potential impacts on IMB skills model constructs will be numerically summarized and graphically visualized (eg, using side-by-side boxplots and scatter plots), as recommended for exploratory data [74].

In-depth interview transcripts will be imported into software for qualitative data analysis (eg, Dedoose [Socio Cultural Research Consultants, LLC]). Thematic analysis will be used to identify, analyze, and report patterns in the qualitative data [75,76]. This approach is being chosen because it allows for flexibility within both postpositivist and constructivist paradigms, which is consistent with mixed methods research [77]. We will follow the six stages of thematic analysis proposed by Braun and Clarke [77]: (1) becoming familiar with the data, (2) generating initial codes, (3) searching for themes, (4) reviewing themes, (5) defining and naming themes, and (6) writing up the results. The trustworthiness and authenticity of the qualitative analysis will be enhanced through multiple mechanisms, including double coding a subset of transcripts independently, conducting regular meetings among the study team members to discuss and resolve discrepancies, and maintaining documentation for auditing purposes.

Integration at the design level will be achieved by our use of a sequential explanatory mixed methods design wherein we will first collect quantitative data on the feasibility and acceptability of our intervention and then collect qualitative data on attitudes, facilitators, and barriers related to engaging in different intervention components. Integration at the methods level will be achieved via (1) connecting (ie, when one form of data links to the other form through the sampling frame), as we will select a purposive subsample of 20 (27%) participants for the qualitative phase from 75 participants in the quantitative phase, and (2) building (ie, when results from the first phase inform data collection in the second phase), as we will use information on participants’ level of engagement in different intervention components to guide our in-depth interviews. Integration at the interpretation level will be achieved through a comparison of our quantitative findings (ie, potential variations in our intervention’s feasibility and acceptability across selected participant characteristics) with our qualitative findings (ie, themes describing attitudes, facilitators, and barriers related to engaging in each intervention component) Integration at the reporting level will be achieved by representing comparisons through combined written descriptions of the quantitative and qualitative data (eg, statistics by themes) and visual representations (eg, joint displays). Results from our study will be contextualized by and compared to the existing literature on home specimen self-collection and telehealth-delivered MI in other sexual and gender minority populations in the United States.

## Results

Our study was funded by the National Institutes of Health in February 2023 and was approved by the Institutional Review Board at the University of Michigan in September 2023. Participant recruitment began in April 2024.

## Discussion

### Principal Findings

Our study is unique with respect to combining home specimen self-collection and return for gonorrhea, chlamydia, and syphilis testing with MI-guided discussions over live AV conferencing, a novel combination that has not yet been explored in GBMSM-LWH. Thus far, most MI-based interventions for people living with HIV have been delivered in person [45-47,78-80], and few have prioritized GBMSM [45-47]. Our pretest sessions focus on eliciting awareness of bacterial STIs; filling any knowledge gaps; bolstering the perceived importance of regularly testing for gonorrhea, chlamydia, and syphilis; and improving self-efficacy for specimen self-collection. Our posttest sessions focus on preparing participants to receive test results and formulating personalized action plans for seeking treatment (if warranted) and repeat testing. Our findings will help fill a void in existing knowledge on the feasibility and acceptability of using a synchronous telehealth modality to deliver MI for engaging sexually active GBMSM-LWH in bacterial STI screening.

### Strengths and Limitations

Combining quantitative and qualitative approaches offers several advantages, such as complementing each other’s strengths and weaknesses, reducing biases, and allowing for the triangulation and contextualization of results [81,82]. Our use of a sequential explanatory mixed methods design will provide a more nuanced understanding of which intervention components might benefit from enhancements to suit the varying needs of GBMSM-LWH in the United States. Recruiting participants via advertising on mobile dating apps and social networking sites can limit generalizability [83], but prior research indicates that GBMSM recruited via the web have a similar prevalence of HIV, bacterial STIs, and screening [84] and a higher engagement in sexual risk behaviors [85,86] compared to those recruited via time-location sampling. We recognize practical issues such as low survey response rates, scheduling conflicts for the pretest and posttest sessions, and the potential for the nondelivery of specimen self-collection boxes due to incorrect mailing addresses, but these have not been problematic in our previous studies among people without HIV that involved similar procedures [28-30,87-89]. If we face challenges in scheduling a posttest session, we will ensure the accessibility of bacterial STI test results in a timely manner via a secure Dropbox folder. Although it might be difficult to re-engage participants who completed only a subset of the 3 intervention components (in phase 1) for in-depth interviews (in phase 2), we hope the incentive being offered for their time will help circumvent this obstacle while being noncoercive. Finally, we acknowledge that assessing the adequacy of specimens for laboratory testing is dependent upon their return.

### Dissemination Plan

Our study’s findings will be disseminated through publications in peer-reviewed journals, presentations at academic conferences, and posts on social media.

### Conclusions

Our study will advance multiple goals of the STI National Strategic Plan for the United States for 2021 to 2025, specifically those pertaining to preventing new STIs; accelerating progress in STI research, technology, and innovation; and reducing STI-related health disparities [90]. Our findings will guide refinements to our intervention before the assessment of its potential impact on improving gonorrhea, chlamydia, and syphilis screening rates among GBMSM-LWH in a future full-scale clinical trial.

## Appendix

Multimedia Appendix 1 Peer-review report: clinical management in general care settings study section, health care return and methodologies integrated review group, Center for Scientific Review, National Institutes of Health.

## Abbreviations

ART: antiretroviral therapy
AV: audio and video
CAPTCHA: Completely Automated Public Turing test to tell Computers and Humans Apart
CDC: Centers for Disease Control and Prevention
GBMSM-LWH: gay, bisexual, and other men who have sex with men living with HIV
IMB: information-motivation-behavioral
MI: motivational interviewing
STI: sexually transmitted infection

## Data Availability

Data sharing is not applicable to this manuscript, as no data sets have been generated or analyzed at this stage of the study. Once analyses related to the study’s objectives have been completed, the data sets will be available from the corresponding author upon reasonable request.
