# Supplementary material for: Feasibility and Acceptability of a Motivational Interviewing–Based Telehealth Intervention for Bacterial Sexually Transmitted Infection Screening: Protocol for a Sequential Explanatory Mixed Methods Study
Source: JMIR Res Protoc. 2024 Aug 29;13:e64433. doi: 10.2196/64433 (PMC11393502; doi:10.2196/64433)
Supplement: Multimedia Appendix 1 [file resprot_v13i1e64433_app1.pdf]

SUMMARY STATEMENT
(Privileged Communication)

PROGRAM CONTACT:
ELEANORE Chuang

Release Date: **11/04/2022**
Revised Date:

Application Number: **1 R21 AI168606-01A1**
Formerly: **1R21AI168606-01**

Principal Investigator

SHARMA, AKSHAY

Applicant Organization: UNIVERSITY OF MICHIGAN AT ANN ARBOR

Review Group: **CMGC**
Clinical Management in General Care Settings Study Section

Meeting Date: **10/17/2022**
Council: **JAN 2023**
Requested Start: **04/01/2023**
RFA/PA: **PA20-194**
PCC: **M37B A**

Project Title: **Feasibility and Acceptability of an MI-based Telehealth Intervention for Bacterial STI Screening**
SRG Action: **Impact Score:10**
Next Steps: **Visit https://grants.nih.gov/grants/next_steps.htm**
Human Subjects: 30-Human subjects involved - Certified, no SRG concerns
Animal Subjects: 10-No live vertebrate animals involved for competing appl.
Gender: 3A-Only men, scientifically acceptable
Minority: 1A-Minorities and non-minorities, scientifically acceptable
Age: 3A-No children included, scientifically acceptable

Project Year	Direct Costs Requested	Estimated Total Cost
1	150,000	234,000
2	125,000	195,000
TOTAL	275,000	429,000

ADMINISTRATIVE BUDGET NOTE: The budget shown is the requested budget and has not been adjusted to reflect any recommendations made by reviewers. If an award is planned, the costs will be calculated by Institute grants management staff based on the recommendations outlined below in the COMMITTEE BUDGET RECOMMENDATIONS section.

1R21AI168606-01A1 Sharma, Akshay

RESUME AND SUMMARY OF DISCUSSION: This application proposes to investigate the feasibility and acceptability of a motivational interviewing-based telehealth intervention to engage gay and bisexual men who have sex with men living with HIV (GBMSM-LWH) in screening for sexually transmitted infections (STIs). Reviewers agreed this project is highly significant given the target population has a high need of screening based on the high rates of bacterial STIs. Additionally, the rigor of the prior research is well articulated to support the development of this intervention for this population. The resubmission is highly responsive and prior minor scoring driving concerns have been remedied, including experience with recruiting from online sources and engaging participants in at-home self-testing. This project is led by an outstanding research team with the appropriate expertise in STI self-testing, motivational interviewing, and qualitative research. Strengths in the approach include the proposed recruitment plan reflects racial and ethnic distribution of GBMSM-LWH in the United States. Rigorous and validated measures of feasibility and acceptability are described. Prior research by the Principal Investigator enrolled a diverse sample of GBMSM without HIV through social media and had a high rate of participation with this approach, demonstrating its success as a recruitment strategy. The review panel only discussed one very minor and addressable weakness, that although the combination of integrating home specimen self-collection with motivational interview guided discussions over video conferencing is innovative, individually the components lack innovation. Overall, this is a highly significant project led by an outstanding research team that will have a high impact on the development of a self-testing intervention for sexually transmitted infection screenings among gay and bisexual men who have sex with men living with HIV.

DESCRIPTION (provided by applicant): Gay, bisexual and other men who have sex with men living with HIV (GBMSM-LWH) bear a heavy burden of bacterial sexually transmitted infections (STIs) such as gonorrhea (GC), chlamydia (CT) and syphilis. Left untreated, bacterial STIs may lead to serious health complications. Inflammatory and ulcerative STIs can also facilitate the onward sexual transmission of HIV in the presence of inadequate viral suppression. Timely diagnosis and treatment are key to prevention. Sexually active GBMSM-LWH engaged in HIV medical care are not being screened for GC, CT and syphilis at least annually, as recommended. Home specimen self-collection has increasingly been used to screen for bacterial STIs in studies conducted with diverse populations. Telehealth has also demonstrated promise in managing mental health and increasing antiretroviral therapy adherence in people living with HIV. Only few studies have combined home specimen self-collection with live audio/video (AV) conferencing, all of which have been restricted to people without HIV. None have focused on GBMSM-LWH or incorporated motivational interviewing (MI), a client-centered, strengths-based counseling approach that seeks to support individuals towards positive behavioral change. Integrating home specimen self-collection from different anatomical sites of possible exposure with MI delivered via live AV conferencing might offer a unique solution to engage GBMSM-LWH in bacterial STI screening. MI-guided discussions have the potential to increase participants' knowledge of bacterial STIs, enhance their intrinsic motivation to protect themselves and their sex partners, improve their self-efficacy for specimen self-collection, and problem-solve barriers to seeking treatment (if warranted) and repeat testing. Our sequential explanatory mixed-methods study seeks to explore the feasibility and acceptability of a novel MI-based telehealth intervention for sexually active GBMSM-LWH. In Phase 1, we will recruit 75 participants via mobile dating apps and social networking websites, and deliver a 3- component intervention: (i) a pre-test live AV conferencing session involving an MI-guided discussion to elicit awareness of bacterial STIs and fill any knowledge gaps, bolster the perceived importance of regularly screening for GC, CT and syphilis, and improve self-efficacy for specimen self-collection, (ii) self-collecting at home and returning by mail a finger-stick blood sample (for syphilis testing), a urine sample (for GC and CT testing), a pharyngeal swab (for GC and CT testing) and a rectal swab (for GC and CT testing), and (iii) a post-test live AV conferencing session involving an MI-guided discussion to prepare participants for receiving test results and formulate personalized action plans for seeking treatment (if warranted) and repeat testing. In Phase 2, we will

conduct in-depth interviews with a purposively selected subsample of 20 participants who complete progressively smaller subsets of the pre-test session, specimen return for bacterial STI testing, and the post-test session to elucidate attitudes, facilitators and barriers related to engaging in each component of our intervention.

PUBLIC HEALTH RELEVANCE: Despite an increase in home specimen self-collection for bacterial sexually transmitted infection (STI) screening in studies conducted with diverse populations, and the demonstrated promise of telehealth in managing mental health and improving antiretroviral therapy adherence in people living with HIV, no studies have combined home specimen self-collection with live audio/video (AV) conferencing to deliver motivational interviewing (MI), a client-centered, strengths-based counseling approach. Our sequential explanatory mixed-methods study seeks to explore the feasibility and acceptability of a novel MI-based telehealth intervention to engage sexually active gay, bisexual and other men who have sex with men living with HIV (GBMSM-LWH) in gonorrhea (GC), chlamydia (CT) and syphilis screening. Our intervention is a package of 3 components: (i) a pre-test live AV conferencing session involving an MI-guided discussion to elicit awareness of bacterial STIs and fill any knowledge gaps, bolster the perceived importance of regularly screening for GC, CT and syphilis, and improve self-efficacy for specimen self-collection, (ii) self-collecting at home and returning by mail a finger-stick blood sample (for syphilis testing), a urine sample (for GC and CT testing), a pharyngeal swab (for GC and CT testing) and a rectal swab (for GC and CT testing), and (iii) a post-test live AV conferencing session involving an MI- guided discussion to prepare participants for receiving test results and formulate personalized action plans for seeking treatment (if warranted) and repeat testing.

CRITIQUE 1

Significance: 1
Investigator(s): 1
Innovation: 4
Approach: 1
Environment: 1

Overall Impact: This highly responsive resubmission will investigate the feasibility and acceptability of a novel motivational interviewing (MI)-based telehealth intervention to engage gay and bisexual men who have sex with men living with HIV in GC, CT and syphilis screening. The 2-year sequential explanatory mixed-methods project contains 3 components: a pre-test live AV conference, self-collecting STI testing at home and returning by mail, and a post-test live AV conferencing session involving an MI-guided discussion. This superbly detailed proposal contains strongly positive score driving aspects in all categories. The target population is in need with high rates of bacterial STIs. The work is theoretically grounded. The investigators are exceptionally well qualified with deep experience in expanding STI testing with novel approaches and motivational interviewing in marginalized populations. The team is slim and highly accomplished. The work is innovative combining three approaches: home specimen self-collection of blood, urine, and rectal sampling, motivational interviewing-guided discussions over live AV conferencing. The approach is rigorous using validated measures and over recruitment of minority populations is a further strength. The U of M environment is exceptionally supportive. The prior minor score driving considerations have been overcome as they now experience recruiting from online sources AND engaging those participants in at-home self-testing. This is highly positively score driving. The innovation is limited to the combination of the three study components. Overall, this is a very well designed study that has a high likelihood of advancing the field in an area of need.

1. Significance:

Strengths

- U.S. The incidence of GC, chlamydia, and syphilis in this population is extraordinarily high (7-12% positive testing rate for each) though the proportion of the population getting tested is low.
- Barriers to testing have been identified.
- The Infectious Diseases Society of America supports using telehealth to deliver “up-to-date, timely, cost-effective” care to marginalized populations and motivational interviewing improves health behaviors of those living with HIV but has not been adequately tested in the target population nor to increase bacterial STI testing.
- The rigor or prior research support development of this intervention in this population.

Weaknesses

- None noted by reviewer

2. Investigator(s):

Strengths

- The PI has an established record of first authorship on new approaches to STI testing and self-testing across gender identities. The addition of an established expert in motivational interviewing (for Aim 1) with a history of NIH funding also strongly and favorably impacts the proposal.
- Highlighting the PI experience in leading an NIH-funded mixed-methods study and the Co-I’s history of qualitative research is responsive and positively score driving.

Weaknesses

- None noted by reviewer

3. Innovation:

Strengths

- The combination of integrating home specimen self-collection for syphilis and triple-site GC and CT testing with MI-guided discussions over live AV conferencing is innovative.

Weaknesses

- Individually the components lack innovation.

4. Approach:

Strengths

- The recruited population will mirror the current U.S. racial/ethnic distribution of GBMSM-LWH in the US.
- Support for the data collection approaches (such as self-report of HIV testing and results) is supported by prior work.
- Rigorous and validated measures of feasibility and acceptability are included. The mixed method design seems particularly appropriate for testing an intervention at this level of maturity.
- The addition of specific go/no-go criteria for the Aim 1 findings is responsive and increases the rigor of the work.

- The use of specimen self-collection kits and instructions provided by the same Emory lab used for this project in over a dozen studies including work by these investigators supports feasibility. As does preliminary data from recent NIH-funded studies that successfully implemented online recruitment, home specimen self-collection and telehealth-delivered MI.

Weaknesses

- None noted by reviewer

5. Environment:

Strengths

- The U of Michigan provides extraordinary resources including in this case the Center for Sexuality and Health Disparities within the School of Nursing. STI testing will be sent to the Emory University Clinical Virology Research Lab which is extremely well-equipped to conduct this testing.

Weaknesses

- None noted by reviewer

Study Timeline:

Strengths

- The timeline is appropriate

Weaknesses

- None noted by reviewer

Protections for Human Subjects

Acceptable Risks and/or Adequate Protections

- Extensive precautions are in place to protect the participants and their data.

Data and Safety Monitoring Plan (Applicable for Clinical Trials Only):

Acceptable

- o The investigators will monitor the data for Aes and SAEs and will form a DSMB.

Inclusion Plans

- Sex/Gender: Distribution justified scientifically
- Race/Ethnicity: Distribution justified scientifically
- For NIH-Defined Phase III trials, Plans for valid design and analysis: Not applicable
- Inclusion/Exclusion Based on Age: Distribution justified scientifically
- The target population limits participation to a sub-population of those who identify as men and oversamples for those from racial and ethnic minority groups

Vertebrate Animals

Not Applicable (No Vertebrate Animals)

Biohazards

Not Applicable (No Biohazards)

Resubmission

- This is a highly responsive resubmission with close attention.

Resource Sharing Plans

Acceptable

Budget and Period of Support

Recommend as Requested

CRITIQUE 2

Significance: 1
Investigator(s): 1
Innovation: 1
Approach: 1
Environment: 1

Overall Impact: This is a resubmission of a R21 proposal focused on evaluating the feasibility and acceptability of a telehealth intervention for bacterial STI screening among GBMSM-LWH in the US. This was a well written application with excellent preliminary data from prior work in a similar population, excellent data supporting the research team's success in recruiting from social media platforms, a sound conceptual framework and a highly experienced research team. There were no significant weaknesses in the application. Thus, findings from this research will be sure to have a sustained impact in the field.

1. Significance:

Strengths

- GBMSM-LWH bear a heavy burden of bacterial sexually transmitted infections (STIs) such as gonorrhea (GC), chlamydia (CT) and syphilis. Left untreated, bacterial STIs can result in serious health complications and can facilitate the transmission of HIV without adequate viral suppression.

Weaknesses

- None noted by reviewer

2. Investigator(s):

Strengths

- Dr. Sharma is an infectious disease epidemiologist whose research focuses on engaging vulnerable populations such as GBMSM in HIV and STI prevention activities including the use of telehealth to overcome some of these barriers.
- He has prior research experience in this area as PI on a NIH-funded study (Project Caboodle) evaluating the acceptability and feasibility of specimen self-collection for STIs among high-risk populations.
- Dr. Bonar is a psychologist who has extensive clinical training in MI and has research expertise in qualitative methods.

Weaknesses

- None noted by reviewer

3. Innovation:

Strengths

- Home specimen self-collection for GC, CT and syphilis testing combined with MI-guided education and discussions over live AV conferencing brings innovation to the proposal.
- Evaluated the feasibility of this combined intervention in the population of sexually active GBMSM-LWH is also highly significant and innovative.

Weaknesses

- None noted by reviewer

4. Approach:

Strengths

- In Project Caboodle, PI Sharma enrolled a diverse sample of 100 GBMSM without HIV via Grindr and Facebook (63% were racial/ethnic minorities): 51% of participants returned self-collected specimens for HIV, GC, CT and potential PrEP adherence testing in ≤6 weeks without MI, incentives or reminders. In other studies, they enrolled 81 GBMSM-LWH in 10 days and 147 GBMSM-LWH in 4 weeks solely via Facebook.
- Emails and mobile phone numbers will be verified via required response to a test email and text. Individuals who do not consent, do not meet the eligibility criteria or do not provide valid contact information will be excluded and directed to the CDC’s website on STI prevention.
- Survey constructs and measures were appropriate and described in detail.
- Each intervention component (1-3) was well described.
- For participants receiving a positive GC, CT or syphilis test result, the clinical pathways that will occur to provide them and their partner with timely treatment were described.
- All videoconferencing sessions will be audio recorded to assess MI fidelity.
- Pilot outcomes (i.e., intervention feasibility, acceptability, knowledge, STI prevalence, receipt of treatment) were well described and appropriate for the proposed research.
- Purposive sampling will be used for qualitative interviews to select a mix of participants who complete progressively smaller subsets of the pre-test session, specimen return for bacterial STI testing, and the post-test session.

- Approach to qualitative analysis is appropriate and integration of qualitative and quantitative findings is well described.

Weaknesses

- None noted by reviewer

5. Environment:

Strengths

- The University of Michigan is home to the Center for Sexuality and Health Disparities which offers resources specific to sexuality research. They will support the qualitative components of the research.
- Investigators will partner with the Emory University Clinical Virology Research Lab (CVRL) which will provide self-collection kits for the study and analyze the samples. This lab is CLIA-certified so results can be used to determine treatment needs.

Weaknesses

- None noted by reviewer

Study Timeline:

Strengths

- Appropriate

Weaknesses

- None noted by reviewer

Protections for Human Subjects

Acceptable Risks and/or Adequate Protections

- None

Data and Safety Monitoring Plan (Applicable for Clinical Trials Only):

Acceptable

- none

Inclusion Plans

- Sex/Gender: Distribution justified scientifically
- Race/Ethnicity: Distribution justified scientifically
- For NIH-Defined Phase III trials, Plans for valid design and analysis: Not applicable
- Inclusion/Exclusion Based on Age: Distribution justified scientifically
- None

Vertebrate Animals

Not Applicable (No Vertebrate Animals)

Biohazards

Not Applicable (No Biohazards)

Resubmission

- The application was highly responsive to the prior critiques.

Resource Sharing Plans

Not Applicable (No Relevant Resources)

Budget and Period of Support

Recommend as Requested

CRITIQUE 3

Significance: 2
Investigator(s): 1
Innovation: 3
Approach: 2
Environment: 1

Overall Impact: This is a resubmitted application proposes to use a mixed-methods approach to examine the feasibility and acceptability of a motivational interviewing intervention to promote better STI screening in GBMSM-LWH. This is a well-written proposal and a significant area of study. The research team is strong and experienced. Prior critiques were positive with some concerns that have been addressed by the investigators. Increased attention to and plans for providing answers to specific participant concerns is an added strength. Overall, the proposed work is likely to have a high impact.

1. Significance:

Strengths

- Addressing known barriers to STI screening.
- Theory based proposal.
- Use of MI, a known successful technique.

Weaknesses

- No significant weaknesses.

2. Investigator(s):

Strengths

- The PI has a strong track record in work proposed.
- The supporting team has the necessary expertise to successfully complete this project.

Weaknesses

- None noted by reviewer.

3. Innovation:

Strengths

- The home-based screening of STI with proposed intervention is innovative.

Weaknesses

- In general, study design and intervention approaches are appropriate though have been used in other settings/populations.

4. Approach:

Strengths

- Mixed methods design.
- Sample size is justified.
- Surveys are appropriate to study questions.

Weaknesses

- Intervention may not address all barriers to screening.

5. Environment:

Strengths

- Exceptionally strong environment (Emory and Michigan) for the proposed work.

Weaknesses

- None noted by reviewer.

Study Timeline:

Strengths

- Timeline is appropriate.

Weaknesses

- None noted by reviewer.

Protections for Human Subjects

Acceptable Risks and/or Adequate Protections

- Acceptable.

Data and Safety Monitoring Plan (Applicable for Clinical Trials Only):

Acceptable

- Acceptable

Inclusion Plans

- Sex/Gender: Distribution justified scientifically

- Race/Ethnicity: Distribution justified scientifically
- For NIH-Defined Phase III trials, Plans for valid design and analysis:
- Inclusion/Exclusion Based on Age: Distribution justified scientifically

Vertebrate Animals

Not Applicable (No Vertebrate Animals)

Biohazards

Not Applicable (No Biohazards)

Resubmission

- Critiques from prior review have been adequately addressed.

Resource Sharing Plans

Not Applicable (No Relevant Resources)

Budget and Period of Support

Recommend as Requested

Recommended budget modifications or possible overlap identified:

THE FOLLOWING SECTIONS WERE PREPARED BY THE SCIENTIFIC REVIEW OFFICER TO SUMMARIZE THE OUTCOME OF DISCUSSIONS OF THE REVIEW COMMITTEE, OR REVIEWERS' WRITTEN CRITIQUES, ON THE FOLLOWING ISSUES:

PROTECTION OF HUMAN SUBJECTS: ACCEPTABLE

INCLUSION OF WOMEN PLAN: ACCEPTABLE

INCLUSION OF MINORITIES PLAN: ACCEPTABLE

INCLUSION ACROSS THE LIFESPAN: ACCEPTABLE

COMMITTEE BUDGET RECOMMENDATIONS: The budget was recommended as requested.

Footnotes for 1 R21 AI168606-01A1; PI Name: Sharma, Akshay

NIH has modified its policy regarding the receipt of resubmissions (amended applications).See Guide Notice NOT-OD-18-197 at https://grants.nih.gov/grants/guide/notice-files/NOT-OD-18-197.html. The impact/priority score is calculated after discussion of an application by averaging the overall scores (1-9) given by all voting reviewers on the committee and multiplying by 10. The criterion scores are submitted prior to the meeting by the individual reviewers assigned to an application, and are not discussed specifically at the review meeting or calculated into the overall impact score. Some applications also receive a percentile

ranking. For details on the review process, see
http://grants.nih.gov/grants/peer_review_process.htm#scoring.

MEETING ROSTER

Clinical Management in General Care Settings Study Section
Healthcare Delivery and Methodologies Integrated Review Group
CENTER FOR SCIENTIFIC REVIEW
CMGC
10/17/2022 - 10/18/2022

Notice of NIH Policy to All Applicants: Meeting rosters are provided for information purposes only. Applicant investigators and institutional officials must not communicate directly with study section members about an application before or after the review. Failure to observe this policy will create a serious breach of integrity in the peer review process, and may lead to actions outlined in NOT-OD-22-044 at https://grants.nih.gov/grants/guide/notice-files/NOT-OD-22-044.html, including removal of the application from immediate review.

CHAIRPERSON(S)

BADGER, TERRY A, RN, PHD
PROFESSOR
COLLEGE OF NURSING
UNIVERSITY OF ARIZONA
TUCSON, AZ 85721

MEMBERS

BECK, AMY LAURA, MPH, MD *
ASSOCIATE PROFESSOR
SCHOOL OF MEDICINE
UNIVERSITY OF CALIFORNIA
SAN FRANCISCO, CA 94116

BILLIMEK, JOHN, PHD
ASSOCIATE PROFESSOR
DEPARTMENT OF FAMILY MEDICINE
SCHOOL OF MEDICINE
UNIVERSITY OF CALIFORNIA, IRVINE
IRVINE, CA 92617

BRODER-FINGERT, SARABETH, MD *
DIRECTOR
DEPARTMENT OF PEDIATRICS
UMASS MEMORIAL MEDICAL CENTER
WORCESTER, MA 01655

EAKIN, MICHELLE NUTTALL, PHD
ASSOCIATE PROFESSOR
DEPARTMENT OF PULMONARY AND CRITICAL CARE MEDICINE
SCHOOL OF MEDICINE
JOHNS HOPKINS UNIVERSITY
BALTIMORE, MD 21224

FAN, VINCENT S, MPH, MD
ASSOCIATE PROFESSOR
DEPARTMENT OF MEDICINE
UNIVERSITY OF WASHINGTON
SEATTLE, WA 98195

HAQUE, REINA, MPH, PHD
RESEARCH SCIENTIST III & PROFESSOR
DEPARTMENT OF RESEARCH AND EVALUATION
KAISER PERMANENTE SOUTHERN CALIFORNIA
PASADENA, CA 91101

KRANS, ELIZABETH E, MD
ASSOCIATE PROFESSOR
DEPARTMENT OF OBSTETRICS, GYNECOLOGY AND REPRODUCTIVE SCIENCES
MAGEE-WOMEN'S HOSPITAL
UNIVERSITY OF PITTSBURGH MEDICAL CENTER
PITTSBURGH, PA 15213

KUNIN-BATSON, ALICIA S, PHD *
ASSISTANT PROFESSOR
DEPARTMENT OF PEDIATRICS
UNIVERSITY OF MINNESOTA
MINNEAPOLIS, MN 55414

LEBARON, VIRGINIA TOWNSEND, PHD *
ASSOCIATE PROFESSOR
SCHOOL OF NURSING
UNIVERSITY OF VIRGINIA
CHARLOTTESVILLE, VA 22908

LEVIN, JENNIFER BETH, PHD
PROFESSOR
DEPARTMENT OF PSYCHIATRY
SCHOOL OF MEDICINE
CASE WESTERN RESERVE UNIVERSITY
CLEVELAND, OH 44106

LIN, JENNY J, MPH, MD *
PROFESSOR
DEPARTMENT OF INTERNAL MEDICINE
ICAHN SCHOOL OF MEDICINE AT MOUNT SINAI
NEW YORK, NY 10029

MCELFISH, PEARL, PHD *
ASSOCIATE PROFESSOR
DEPARTMENT OF INTERNAL MEDICINE
UNIVERSITY OF ARKANSAS
FAYETTEVILLE, AR 72703

MILITELLO, LISA KINSELLA, PHD *
ASSISTANT PROFESSOR
COLLEGE OF NURSING
OHIO STATE UNIVERSITY
COLUMBUS, OH 43210

NELSON, LONNIE A, PHD
ASSOCIATE PROFESSOR
COLLEGE OF NURSING
WASHINGTON STATE UNIVERSITY
SPOKANE, WA 99202

PEREZ-LOUGEE, GISELLE KATIRIA, PHD *
ASSISTANT PROFESSOR
DEPARTMENT OF PSYCHIATRY
HARVARD MEDICAL SCHOOL
BOSTON, MA 02114

PIATT, GRETCHEN A, MPH, PHD
ASSOCIATE PROFESSOR
DEPARTMENT OF LEARNING HEALTH SCIENCES
UNIVERSITY OF MICHIGAN
ANN ARBOR, MI 48109

PICKERING, CAROLYN E, RN, PHD
ASSOCIATE PROFESSOR
SCHOOL OF NURSING
UNIVERSITY OF ALABAMA, BIRMINGHAM
BIRMINGHAM, AL 35226

PORTZ, JENNIFER, PHD *
ASSISTANT PROFESSOR
DEPARTMENT OF INTERNAL MEDICINE
UNIVERSITY OF COLORADO DENVER
AURORA, CO 80045

QUINONES, ANA ROMAN, PHD
ASSOCIATE PROFESSOR
DEPARTMENT OF FAMILY MEDICINE
SCHOOL OF MEDICINE
OREGON HEALTH AND SCIENCE UNIVERSITY
PORTLAND, OR 97239

SOMERS, TAMARA J, PHD
ASSOCIATE PROFESSOR
DEPARTMENT OF PSYCHIATRY AND BEHAVIORAL SCIENCES
DUKE UNIVERSITY
DURHAM, NC 27705

SONG, MI-KYUNG, FAAN, PHD
PROFESSOR
NELL HODGSON WOODRUFF SCHOOL OF NURSING
EMORY UNIVERSITY
ATLANTA, GA 30322

THOMPSON, TESS, MPH, PHD *
ASSISTANT PROFESSOR
DEPARTMENT OF PUBLIC HEALTH
WASHINGTON UNIVERSITY
ST LOUIS, MO 63130

TORKE, ALEXIA M, MD
PROFESSOR
CENTER OF AGING RESEARCH
INDIANA UNIVERSITY
INDIANAPOLIS, IN 46202

TUROK, DAVID, MD
ASSOCIATE PROFESSOR
DEPARTMENT OF OBSTETRICS AND GYNECOLOGY
UNIVERSITY OF UTAH
SALT LAKE CITY, UT 84132

VAN SCOY, LAUREN JODI, MD
ASSOCIATE PROFESSOR
DEPARTMENT OF HUMANITIES AND PULMONARY MEDICINE
COLLEGE OF MEDICINE
THE PENNSYLVANIA STATE UNIVERSITY
HERSHEY, PA 17033

WILLIAMS, KRISTINE N, PHD
PROFESSOR
SCHOOL OF NURSING
UNIVERSITY OF KANSAS MEDICAL CENTER
KANSAS CITY, KS 66160

ZHU, HONG, PHD
ASSOCIATE PROFESSOR
DEPARTMENT OF POPULATION AND DATA SCIENCES
UNIVERSITY OF TEXAS SOUTHWESTERN MEDICAL CENTER
DALLAS, TX 75390

SCIENTIFIC REVIEW OFFICER

FORDYCE, LAUREN, PHD
SCIENTIFIC REVIEW OFFICER
CENTER FOR SCIENTIFIC REVIEW
NATIONAL INSTITUTES OF HEALTH
BETHESDA, MD 20892

* Temporary Member. For grant applications, temporary members may participate in the entire meeting or may review only selected applications as needed.

Consultants are required to absent themselves from the room during the review of any application if their presence would constitute or appear to constitute a conflict of interest.
